# Development of a biofilm inhibitor molecule against multidrug resistant *Staphylococcus aureus* associated with gestational urinary tract infections

**DOI:** 10.3389/fmicb.2015.00832

**Published:** 2015-08-11

**Authors:** P. Balamurugan, M. Hema, Gurmeet Kaur, V. Sridharan, P. C. Prabu, M. N. Sumana, S. Adline Princy

**Affiliations:** ^1^Quorum Sensing Laboratory, Centre for Research on Infectious Diseases, School of Chemical and Biotechnology, SASTRA UniversityThanjavur, India; ^2^Department of Chemistry, Centre for Research on Infectious Diseases, School of Chemical and Biotechnology, SASTRA UniversityThanjavur, India; ^3^Central Animal Facility, SASTRA UniversityThanjavur, India; ^4^Department of Microbiology, JSS Medical College and JSS UniversityMysore, India

**Keywords:** uropathogen, *Staphylococcus aureus*, quorum sensing, biofilm, *Melia dubia*, hybrid molecule, multidrug resistance

## Abstract

Urinary Tract Infection (UTI) is a globally widespread human infection caused by an infestation of uropathogens. Eventhough, *Escherichia coli* is often quoted as being the chief among them, *Staphylococcus aureus* involvement in UTI especially in gestational UTI is often understated. Staphylococcal accessory regulator A (SarA) is a quorum regulator of *S. aureus* that controls the expression of various virulence and biofilm phenotypes. Since SarA had been a focussed target for antibiofilm agent development, the study aims to develop a potential drug molecule targeting the SarA of *S. aureus* to combat biofilm associated infections in which it is involved. In our previous studies, we have reported the antibiofilm activity of SarA based biofilm inhibitor, (SarABI) with a 50% minimum biofilm inhibitory concentration (MBIC_50_) value of 200 μg/mL against *S. aureus* associated with vascular graft infections and also the antibiofilm activity of the root ethanolic extracts of *Melia dubia* against uropathogenic *E. coli*. In the present study, *in silico* design of a hybrid molecule composed of a molecule screened from *M. dubia* root ethanolic extracts and a modified SarA based inhibitor (SarABI^M^) was undertaken. SarABI^M^ is a modified form of SarABI where the fluorine groups are absent in SarABI^M^. Chemical synthesis of the hybrid molecule, 4-(Benzylamino)cyclohexyl 2-hydroxycinnamate (henceforth referred to as UTI Quorum-Quencher, UTI^QQ^) was then performed, followed by *in vitro* and *in vivo* validation. The MBIC_50_ and MBIC_90_ of UTI^QQ^ were found to be 15 and 65 μg/mL, respectively. Confocal laser scanning microscopy (CLSM) images witnessed biofilm reduction and bacterial killing in either UTI^QQ^ or in combined use of antibiotic gentamicin and UTI^*QQ*^. Similar results were observed with *in vivo* studies of experimental UTI in rat model. So, we propose that the drug UTI^QQ^ would be a promising candidate when used alone or, in combination with an antibiotic for staphylococcal associated UTI.

## Introduction

Urinary Tract Infections (UTIs) are among the most common infections that necessitate a hospital visit; some estimates claim UTI to be the second most common infection after the common cold, and being the primary cause of over eight million annual hospital appointments (Schappert and Rechtsteiner, 2008). The infection can be asymptomatic where there is no apparent indication of the infection, or can degenerate into a symptomatic version, with the usual symptoms being frequent and/or painful urination accompanied with abdominal pain and cloudy or bloody rancid-smelling urine. Fever during UTI is often indicative of pyelonephritis, a condition where the infection has reached the kidneys and requiring immediate medical attention. Kidney UTIs are deadly in the case where it effects septicemia, leading to infection in the bloodstream, and can cause systemic infection. Women are more prone to UTIs than men. Several factors are responsible for this—shorter urinary tract, proximity of the urethra to the anal opening, hormonal imbalances and fluctuations leading to pH rise in urethra and use of contraceptives and spermicides are among the prime causes (Stamm and Raz, 1999). About 5% of pregnant women develop UTI (Sharma et al., 2009) and they experience urine retention and urinary reflux that leaves the urothelium vulnerable to infection. The infection, untreated, leads to premature births, morbidity, and mortality in pregnant women (Warren et al., 1999). Gestational UTI is also identified to be the cause of 27% of miscarriages and premature births in a study involving mice (Kaul et al., 1999). Further, risks of pyelonephritis increase with pregnancy. Problems concerning UTI during pregnancy include the non-availability of first-line antibiotics like fluoroquinolones and trimethoprim, primarily because of the side-effects to fetal development (Jancel and Dudas, 2002; Lee et al., 2008).

An assortment of microorganisms act as perpetrators of UTI and *Escherichia coli* is the primary organism to cause more than 80% of UTI. Other organisms responsible are *Staphylococci, Klebsiella, Proteus mirabilis, Enterococcus, Pseudomonas aeruginosa*, and *Serratia marcescens*. Of the fungal causatives, *Candida* sp. are most prominent to complicate UTIs that involves *Candida* “yeast infections” and coagulase-positive *Staphylococci* that may lead to septicemia ultimately. The role of *Staphylococcus aureus* is often understated and there is an increasing prevalence of *S. aureus* in UTI in recent years (Akortha and Ibadin, 2008). In the earlier studies, *S. aureus* was found to be the second most prevalent pathogen in UTI, and it is of higher incidence in women (Onanuga and Awhowho, 2012). It is known to cause 3–6% of UTIs, and up to 25% of UTIs in some cases (Wasnik and Tumane, 2013). Adherence of bacteria to the uroepithelial tissues is important for ascending infection, and hence the formation of biofilm-like communities within the urinary bladder complicates treatment. Also the multidrug resistance (MDR) is associated with biofilm formation in several bacteria, increases the inability of antibiotics to penetrate biofilms playing a key role in such behavior. Most of the virulence factors are expressed by the pathogens during an infection, in tandem, coordinated by a signaling mechanism termed as quorum sensing.

Quorum sensing in Staphylococci is usually mediated by two pathways namely the staphylococcal accessory regulator (*sar*) and accessory global regulator (*agr*) cascades. The *agr* pathway is a more specific pathway that regulates quorum sensing in the pathogen, involving the secretion of a signal peptide extracellularly, and its recognition leading to the expression of virulence factors. The *sarA* locus is more of a global control cascade, controlling the manifestation of many virulence genes, with its mutation known to affect 120 genes, with 76 of them positively regulated and 44 genes negatively regulated (Dunman et al., 2001). The SarA family of proteins includes SarR, SarS, SarT, SarU, SarV, and MgrA, all of which share sequence homology (Cheung et al., 2004; Manna et al., 2004). Mutation of *sarA* effects out reduced biofilm production capabilities, regardless of the status of *agr* cascade expression (Beenken et al., 2010). Moreover, the role of SarA in agr-independent expression of several other virulence genes like activation of *fnbA* (fibronection binding protein A) and *bap* (biofilm associated proteins) had been explained in the previous studies (Trotonda et al., 2005; Roberts et al., 2006). In our previous study, we have experimentally proved that targeting SarA inhibition would be an effective way of inhibiting the biofilm (Arya et al., 2015).

*Melia dubia* which is called as malai vembu indigenously is a plant of many wonderful medicinal properties. Belonging to the Meliaceae family of plants it is found at an elevation of 600–1800 m in the South Indian Ghats, India. Its leaves, barks and fruits have insect repellant properties. The leaf of this tree has essential oils constituted by monoterpene camphene, α-pinene, β-pinene, and sabinene, are is rich in antimicrobial, antiviral, and antineoplastic activity (Nagalakshmi et al., 2003). There has also been successful preventive therapy using infusions of *M. dubia* in Tamil Nadu, India against dengue virus. The methanolic fraction of this plant has anti-larval properties (Koul et al., 2000).

The present study involves the screening, synthesis and validation of a potent compound against SarA of *S. aureus* associated with gestational urinary tract infections, in light of our previous studies that showed potent antibiofilm activity in root ethanolic extract of *M. dubia* against uropathogenic *E. coli* (Vinothkannan et al., 2013; Adline Princy et al., 2014) and antibiofilm activity of SarABI against *S. aureus* associated with vascular graft infections (Arya et al., 2015). In brief, the proposed inhibitor, UTI^QQ^ against SarA protein was computationally validated to have a high affinity toward the target and it was synthesized by a two step process, i.e., reductive amination and steglich esterification for further *in vitro* and *in vivo* validation.

## Materials and methods

### Strains

Urine samples (*N* = 100) were collected at random from the pregnant women during their pre-natal visit at Mother and Child Care Maternity Hospital, Thanjavur between May 2013 and July 2013 and the status of infection and pyuria was analyzed by centrifugation at 6000 rpm, 5 min, followed count of pathogens by the colony count. Pathogenic strains from the confirmed cases causing the urinary tract infections were isolated. The strains were then screened for MDR against a variety of antibiotics used for conventional UTI treatment. The isolated strains (Table 1) were cultured in enriched tryptic soy broth and used for the subsequent study.

**Table 1:** **Strains used in this study**.

**Strain**	**Identifier**	**References**
*Staphylococcus aureus* reference ATCC	ATCC25923	Levy et al., 2003
*Staphylococcus aureus sarA* mutant (Δ*s*arA::Tn917LTV1)	ALC637	Wolz et al., 2000
*Escherichia coli* clinical isolate	EC67a	Princy et al., 2014
*Staphylococcus aureus* clinical isolate (MRSA)	SA13a	Princy et al., 2014
*Pseudomonas aeruginosa* clinical isolate	PA07c	Princy et al., 2014
*Klebsiella pneumonia* clinical isolate	KP32b	Princy et al., 2014
*Enterococcus faecalis* clinical isolate	EF32e	Princy et al., 2014

### Extraction of plant material and biofilm assay

The *M. dubia* root samples collected from Thanjavur, Tamil Nadu were subjected to the process of extraction at room temperature (30 ± 1°C), through cold percolation methods using different solvents (water, hexane, petroleum ether, ethanol, and methanol) in the proportion of 100 g powdered root in 900 mL solvent. The mixture was regularly stirred for 72 h after which the supernatant was recovered by filtration and the solvent in the supernatant was evaporated in a rotary evaporator. The crude extract was lyophilized and stored at −80°C (Ravichandiran et al., 2012). The antibiofilm efficacy of *M. dubia* extracts of different concentrations was observed using a modified crystal violet method as described (O'Toole and Kolter, 1998). The extracts were dissolved in 1X phosphate buffer saline (1X PBS) for the assay. Since the root ethanolic extracts displayed good antibiofilm activity against *S. aureus*, it was taken for the further studies.

### Gas chromatography mass spectrometry (GC-MS)

GC-MS analysis was carried out for the root ethanolic extract of *M. dubia* using a PerkinElmer Clarus 500 GC-MS system. The program was set at a temperature of 50°C for a duration of 1 min and raised at 10°C/min to 150°C (1 min hold), at 8°C/min to 250°C (1 min hold), at 15°C/min to 300°C (3 min hold). Helium (1 mL/min) was used as carrier gas. The injector temperature was maintained at 280°C and the mass range was 40–450 amu. One microliter of sample dissolved in ethanol was injected into the system. The identification of the compounds was made by comparing their spectra with the National Institute of Standard and Technology (NIST) spectral library.

### Computational studies

The compounds reported by GC-MS were drawn using ACD Chemsketch™. The energy minimized 3D ligand conformers were prepared using Schrödinger™ LigPrep software. Glide module of Schrödinger was used for the molecular docking analysis. OPLS-2005 force field was utilized to optimize the geometry and for minimization. SarA protein structure 2FNP (Liu et al., 2006), was prepared and the receptor grid was generated encompassing the whole protein, with the centroid of the protein fixed with a grid size of 20 Å. The compounds were also analyzed for their suitability for the use as a drug molecule, based the Lipinski's Rule of Five. Also, the ADMET (Absorption, Distribution, Metabolism, Excretion, Toxicity) parameters predicted by the Topkat module of Accelrys™ Discovery and Quikprop module of Schrödinger™ Suite were taken into consideration for the efficient screen of various compounds with drug-likeness properties.

The candidate molecule *o*-coumaric acid, as identified by the *in silico* techniques had a carboxylate functional group, and had affinity to bind to R84, more than DER box (88, 89, and 90). Our earlier study had established the molecule 4-(2,4-difluorobenzylamino)cyclohexanol, SarABI which bound to E89 and R90 of SarA (Arya and Princy, 2013; Arya et al., 2015). Since, the acid group of the candidate molecule identified was undesirable, an ester linkage between the acid group and the alcohol group of SarABI was proposed to have a hybrid molecule and analyzed by *in silico*. Molecular docking predicted that absence of fluorine groups in the hybrid molecule had better binding affinity to SarA and hence a modified SarABI (SarABI^M^) i.e., without fluorine atoms and *o*-coumaric acid was considered for the chemical synthesis of a hybrid molecule.

### Chemical synthesis of hybrid molecule, UTI^QQ^

**Figure 1:**
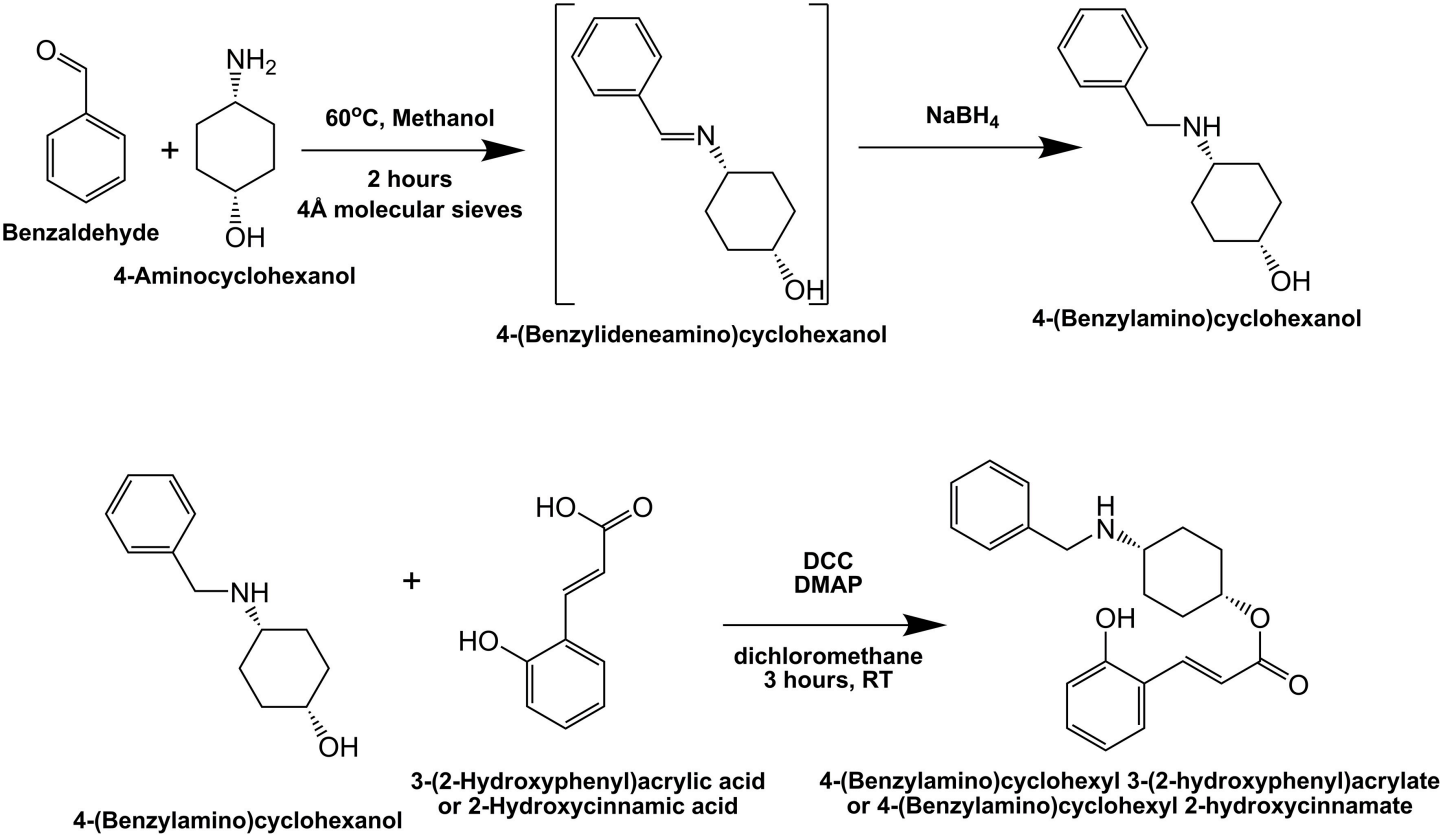
**Synthesis scheme for hybrid molecule, UTI^QQ^**. The compound, 4-(Benzylamino)cyclohexyl 2-hydroxycinnamate (UTI^QQ^) was synthesized in two steps. The first step was reductive amination of Benzaldehyde and 4-aminocyclohexanol. The second step was esterification of 4-(Benzylamino)cyclohexanol and 2-hydroxycinnamic acid/*o*-coumaric acid.

The synthesis is comprised of two parts, reductive amination and esterification (Figure 1). An equimolar mixture of *cis* 4-aminocyclohexanol and benzaldehyde was refluxed in methanol for 3 h at 70°C along with 4 Å molecular sieves. The mixture was cooled using an ice-water bath, and sodium borohydride (l equiv.) was added in small portions. The reaction was then stirred at room temperature overnight. Reaction was quenched by adding cold water and extracted with dichloromethane. The organic layer was washed with NaOH solution, dried over anhydrous sodium sulfate solution and concentrated to obtain the pure 4-(Benzyl amino)cyclohexanol (SarABI^M^). ^1^H-NMR was used to confirm the synthesized compounds.

This was followed by Steglich esterification (Neises and Steglich, 1978). To a solution of SarABI^M^ (0.2 mmol), DMAP (4-Dimethylaminopyridine) (0.2 equiv), and *cis o*-coumaric acid/2-Hydroxycinnamic acid (1.2 equiv.) in anhydrous dichloromethane (8 mL), was added DCC (N,N′-Dicyclohexylcarbodiimide) (1.2 equiv.) at 0°C. The mixture was stirred at room temperature until completion of the reaction. The reaction mixture was filtered, and the residue was washed with dichloromethane (2 × 10 mL). The solution was washed with 5% HCl (3 × 30 mL), saturated sodium bicarbonate (3 × 30 mL) and saturated NaCl (3 × 30 mL), respectively. The organic layer was then dried using sodium sulfate (anhydrous) and vacuum dried. The residue containing the hybrid molecule, 4-(Benzylamino)cyclohexyl 2-hydroxycinnamate (henceforth referred to as UTI quorum-quencher, UTI^QQ^) was used for further assays.

### Determination of minimum biofilm inhibitory concentration (MBIC)

Briefly, 100 μl inoculum of *S. aureus* ATCC 25923 was plated onto polystyrene microtiter plates in the proportion 1:200 (v/v) from an overnight culture. Culturing was done in artificial urine media (Brooks and Keevil, 1997). Varying concentrations of UTI^QQ^ ranging from 1 to 100 μg/mL were added, in triplicates. After 24 h, the planktonic cells were removed. PBS wash was done thrice and the cells were fixed using 100 μL of 99% methanol. Then 150 μL of 0.2% crystal violet was used for 20 min to stain the biofilm cells. Excess stains were removed by washing under slow-flowing cold tap water and the plates were air dried. Again, 33% acetic acid was used to elute the bound crystal violet and the optical reading was read in an ELISA plate reader (BioRad i-Mark, Japan) at 595 nm (Stepanovic et al., 2000). The lowest concentration of the compound that inhibits the biofilm by 50% compared to untreated culture control is the minimum biofilm inhibitory concentration (MBIC_50_) and that by 90% is MBIC_90_.

### Determination of minimum inhibitory concentration (MIC) and minimum bactericidal concentration (MBC)

The minimum inhibitory concentration of a compound is the lowest concentration that retards the visible growth of the microorganism, and is a measure of the cell growth parameters. The minimum bactericidal concentration is the lowest concentration of the compound that causes cell viability loss of 99.9%, i.e., only 1 in 1000 cell survives at MBC of a compound. The cell density was measured at 600 nm before the crystal violet biofilm assay to find the effect of the compound on growth. Further the cells were plated after appropriate serial dilutions of upto 10^8^ X fold, in triplicates, onto the Cation-Adjusted Muller-Hinton Agar (CAMHA) plates. The observed count of the colony forming units per mL of broth (CFU/mL) provides a measure of the survival of the cells. Untreated group was taken as negative control. The experiments for *in vitro* drug response were done twice independently.

### Hydrophobicity assay

18 h old culture (under UTI^QQ^ treatment) in artificial urine media was centrifuged. Mixed cultures of uropathogens were grown in equi-volume ratios. Pellets obtained were washed in PBS and resuspended to an OD_550nm_ = 0.8 (A_0_). Three milliliter of this suspension was mixed with 400 μl of p-xylene, equilibrated in a water bath at 25°C for 10 min and vortexed. The lower aqueous phase optical density was measured at 550 nm (A_1_) (Basson et al., 2008). Measure of hydrophobicity as percentage of adherence to xylene was calculated using the formula given, and then standardized using the untreated control.

$$\begin{array}{l} \%Hydrophobicity=\frac{\left({\text{A}}_{0}-{\text{A}}_{1}\right)}{{\text{A}}_{0}}\times 100 \end{array}$$

### Confocal laser scanning microscopy (CLSM) imaging

Biofilm of mixed bacterial population was developed onto glass cover-slips in a six-well cell culture plate, under various conditions of treatment using SarABI^M^, *o*-coumaric acid, hybrid molecule UTI^QQ^ and antibiotic gentamicin. This was performed to assess qualitatively the effects of the UTI^QQ^ on the biofilm of the mixed population. After the biofilms were grown for 24 h, the suspension was aspirated and removed carefully. The biofilm was rinsed delicately in 0.9% NaCl solution. Stock solutions of the fluorescein isothiocyanate (5 mg/mL) and ethidium bromide (1.25 mg/mL) were prepared beforehand. Five microliter each of the dyes were mixed with 1 mL of cold 0.9% NaCl solution to obtain a working solution. The biofilm was stained with 5 μL of the working solution of the dyes for 10 min, and then the excess dye was removed by washing with 0.9% NaCl. The cover-slip was then dried for 2 min in an ambient temperature and then fixed using 50 μL of toluene. Confocal imaging was performed using Olympus Confocal Laser Scanning Microscope to obtain the live/dead imaging (Netuschil et al., 1989). MIC of gentamicin at 2 μg/mL concentration was used along with the UTI^QQ^ to understand the combinatorial effects.

### Cell culture studies

Hep-G2 cells were seeded in 48 well plate at a seeding density of 15,000 cells/well. The cells were checked for its confluence, once it has attained 70% of confluence, the MBIC_50_ (15 μg/mL) and MBIC_90_(65 μg/mL) concentrations of the synthesized UTI^QQ^ was added. After 24 h of incubation, the supernatant was used for Lactate Dehydrogenase (LDH) assay. The remaining culture medium was analyzed for cell viablity using MTT assay.

### Lactate dehydrogenase (LDH) assay

Cytotoxicity of UTI^QQ^ in its different concentrations was determined by lactate dehydrogenase (LDH) assay. The assay determines the release of cytoplasmic lactate dehydrogenase into the cytosol due to the leakage of the damaged cells. After 24 h of incubation 50 μL of culture medium was collected and incubated with the reaction mixture consisting of NAD^+^ (50 μL), lactate (50 μL), and phosphate buffer (0.2 M) pH 7.4. The absorbance was measured at 340 nm.

### MTT assay

The effect UTI^QQ^ toward Hep-G2 cells was tested using two concentrations, MBIC_50_ and MBIC_90_. The MTT cell viability assay was followed (Zakaria et al., 2009). In brief, sub-confluent HEp-G2 cells were added in the microtitre plate wells along with DMEM medium. After adherence was established in the plates (post 24 h incubation in CO_2_ incubator), UTI^QQ^ was added at an appropriate concentration to the wells, followed by 24 h incubation and addition of MTT i.e., (3-(4,5-Dimethylthiazol-2-yl)-2,5-diphenyltetrazolium bromide) followed an incubation of 3 h with readings were recorded at every 1 h interval. 200 μL of isopropanol was added to the wells and OD was taken at 570 nm.

## *In vivo* studies

### Induction of experimental UTI in pregnant mice by bladder catheterization

Institutional Animal Ethical Committee (IAEC) approval was obtained to conduct the efficacy studies of UTI^QQ^ and antibiotics in experimentally disease induced animal models (IAEC No: 222/SASTRA/IAEC/RPP). Groups of pregnant wistar rats (*Rattus norvegicus*), three rats per group were infected with uropathogenic *S. aureus* on day 7 of pregnancy (32% gestation) by urethral catheterization. Briefly, animals were anesthetized with thiopentone thiosol (40 mg/kg) injection. The bacterial inoculum (0.1 mL of a suspension with an optical density at 600 nm of 0.5) was instilled into the urinary bladder through a soft polyethylene catheter adapted to a needle on a syringe. The control groups received sterile PBS. The rats were placed in individual cages and allowed for free access to food and drink under a 12-h day-night cycle. On the subsequent day, the treatment groups were administered with gentamicin (low dose, 8 mg/kg and high dose, 50 mg/kg) and the hybrid molecule UTI^QQ^ (low dose, 16 mg/kg and high dose, 64 mg/kg). The animals were monitored daily for symptoms like preterm labor, bleeding or any other sickness. Preterm is defined as any delivery occurring on or before day 20 (90% gestation) of gestation (Kaul et al., 1999). The survival of the pups delivered by these rats, either term or preterm, was monitored and subsequent body weights of those pups were also taken.

### Quantitative tissue culture

Quantitative tissue culture was performed in a group of infected rats before completing the delivery and sacrificed on day 17 (77% gestation). Kidney was collected from each rat and homogenized separately in 2.0 mL of sterile PBS. After appropriate dilution, samples were plated on mannitol salt agar (Himedia, Mumbai), for selective isolation of uropathogenic *S. aureus*. The bacterial counts were reported as CFU per gram of the tissues.

### Histological analysis of tissues

Kidney samples collected for the histological analysis were fixed overnight in formalin, embedded in paraffin, sectioned for the immunohistochemical analysis and stained with hematoxylin and eosin. Inflammation grades of 0 to 5 were assigned based on the severity of infection as follows: 0, absent; 1, minimal; 2, mild; 3, moderate; 4, marked; and 5, severe. The overall score for the histopathological grading of pyelonephritis was based on the pelvic, interstitial, and tubular involvement. All the tissue samples were coded and read blind by the observer.

### Statistical analysis

Statistical analysis was carried out using GraphPad prism software version 6.05 (GraphPad Software Inc., SanDiego, CA). One-Way ANOVA followed by multiple comparisons using Tukey's test was used to test the significance. The minimum level of significance was set at *P* ≤ 0.05. All the assays were conducted in triplicates and the values were expressed as mean ± SD.

## Results

### Extraction of plant material and biofilm assay

**Figure 2:**
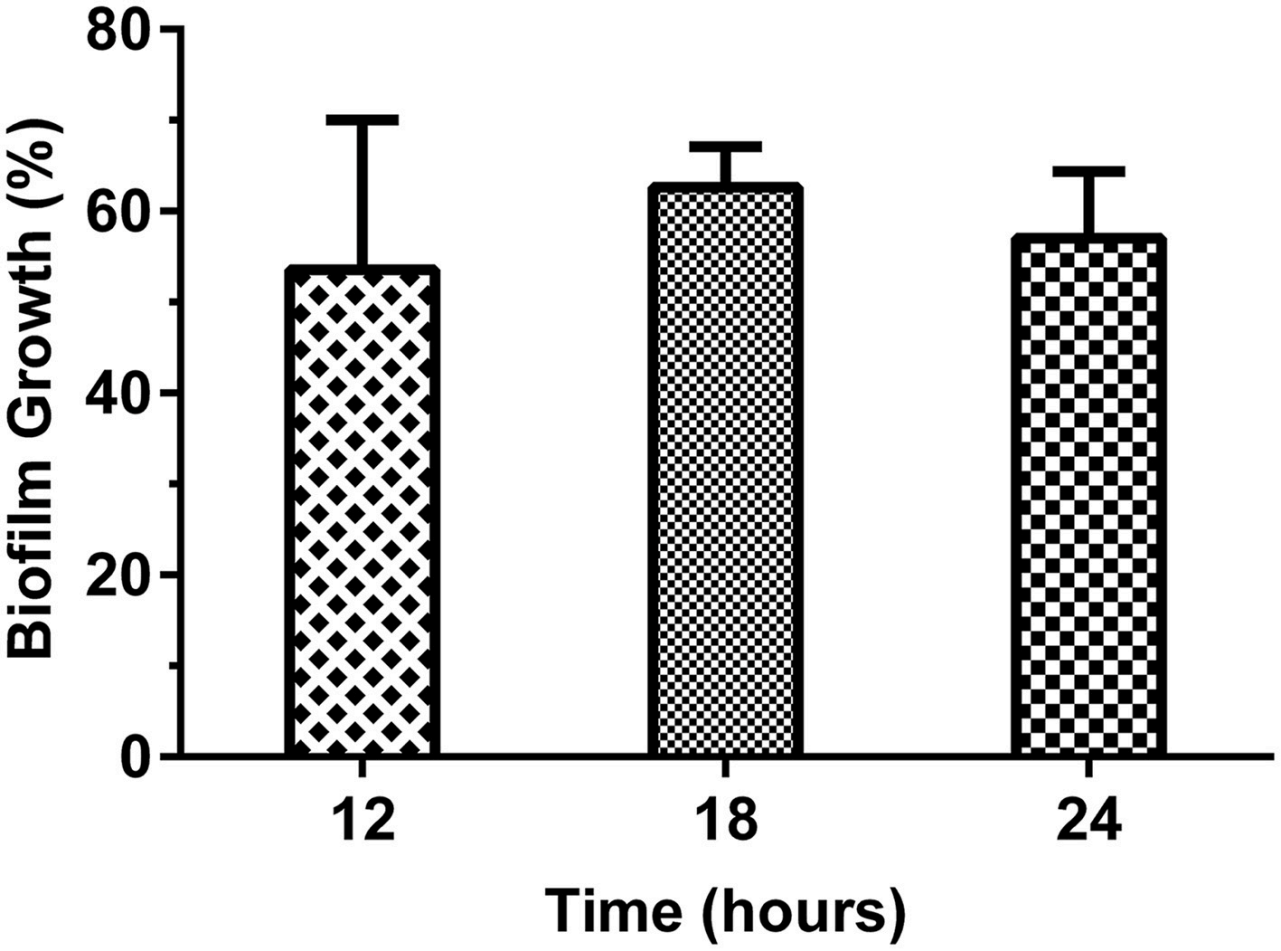
**Determination of ***S. aureus*** ATCC 25923 biofilm formation at different time intervals in response to crude root ethanolic extract of ***M. dubia*** by crystal violet method**. *S. aureus* ATCC 25923 was cultured in tryptic soy broth with different solvent extracts of *M. dubia* in triplicates and biofilm was quantified at different time intervals by crystal violet method. Concentration at which 50% inhibition observed at all time intervals i.e., 40 μg/mL of root ethanolic extract is shown in the figure.

The efficacy of different solvent root extracts of *M. dubia* in suppressing the traits responsible for the establishment of microbial biofilm in ATCC 25923 reference strain of *S. aureus* was tested. Ethanolic extract of the root served to be the best among all the solvent extracts as a biofilm inhibitor. Biofilm formation was retarded by nearly 50% in the presence of 40 μg/mL concentration of the root ethanolic extract (Figure 2).

### Gas chromatography mass spectrometry (GC-MS) and computational studies

The gas chromatography mass spectroscopy (GC-MS) analysis revealed the ingredients of root ethanolic extracts of *M. dubia* (Table S1). The various components were analysed by *in silico* docking simulation that predicts the binding affinity to the SarA protein. The ligands were drawn using Chemsketch software and used consequently for the computational studies. The 40 ligands from the root ethanolic extract of *M. dubia* were pitted against one another, in a bid to successfully unravel the best molecule to proceed with. The top-hit molecules were identified with the drug-likeness properties using the molecular docking software, Schrödinger Suite (Table 2).

**Table 2:** **List of top 10 ligands based of docking G-score with interaction sites and logP-values**.

**S. No**.	**Name of ligand**	**Glide docking score**	**Predicted interaction sites**	**logP**	**Molecular weight (Da)**
1	Sucrose	−6.751	K82, H87, R84, E89	−3.76	342.29
2	Vanillin lactoside	−5.912	K82, R84, D88	−2.29	476.28
3	Ethyl d-glucopyranoside	−5.624	Q64, R84, R90	−2.16	208.21
4	6-Deoxy L-galactose	−5.256	Q64, R84, R90	−2.04	164.16
5	2-hydroxycinnamic acid/*o*-coumaric acid	−4.785	Q64, R84, R90	+2.43	164.16
6	Cyclohexane carboxylic acid, 3-acetyloxyl	−4.441	R90	+0.92	186.20
7	D-mannose	−4.363	Q64, R84, R90	−3.17	180.16
8	1,6 Anhydro D-glucopyranose (levoglycan)	−4.284	K82, R84, D88	−0.04	162.14
9	Benzenepropanol, 4-hydroxyl methyl	−3.761	K54, Q64, R90	+1.49	166.25
10	Dianhydromannitol	−3.642	Q64, R84, R90	−0.25	146.14

**Figure 3:**
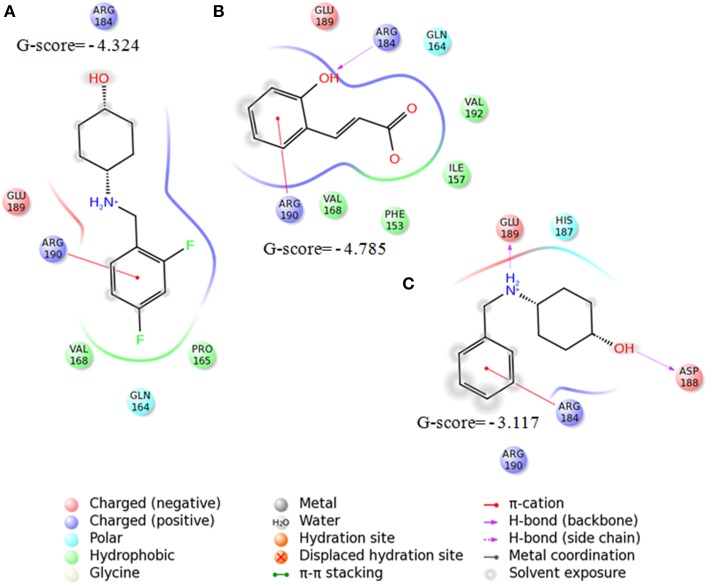
**Interaction patterns of SarABI (A), ***o***-coumaric acid (B), and SarABI^M^ (C) to SarA protein using Schrödinger software**. *o*-coumaric acid has a carboxylate functional group, and had affinity to bind to R84, more than DER box (88, 89, and 90). SarABI bound to E89 and R90 of SarA whereas SarABI^M^ bound to D88 and E89.

**Figure 4:**
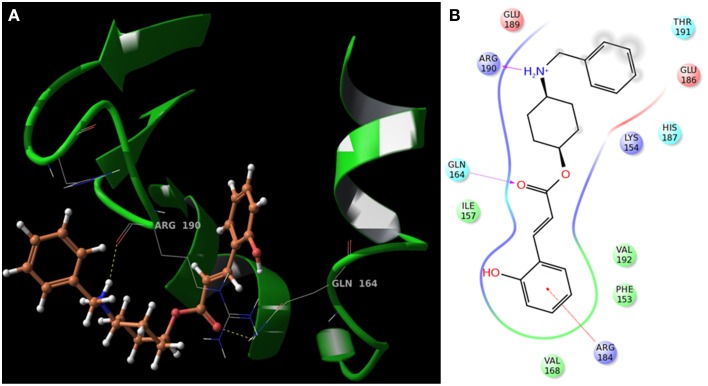
**Molecular docking of UTI^QQ^ to SarA protein using Schrödinger software**. **(A)** Binding pose of UTI^QQ^ to SarA sites, represented by H-bonds at R84 and Q64, **(B)** Interaction diagram also reveals the pi-cation interaction at R84.

The interaction patterns of SarABI, *o*-coumaric acid and SarABI^M^ to SarA protein was shown in Figure 3. *O*-coumaric acid has a carboxylate functional group, and showed higher affinity to interact with the key residues, R84, more than DER box (88, 89, and 90). SarABI bound to E89 and R90 of SarA whereas the SarABI^M^ bound to D88 and E89 with a lesser glide score. The binding pose of UTI^QQ^ to SarA sites, represented by H-bonds at R84 and Q64, and interaction diagram was shown in Figure 4. Molecular docking predicted the absence of fluorine groups in the hybrid molecule, UTI^QQ^ that had a better binding affinity to SarA. ADMET predictions revealed that UTI^QQ^ would have oral absorptivity of 100% and that the LD_50_ in rats would be 3 g/kg.

### Chemical synthesis and *in vitro* validation of UTI^QQ^

**Figure 5:**
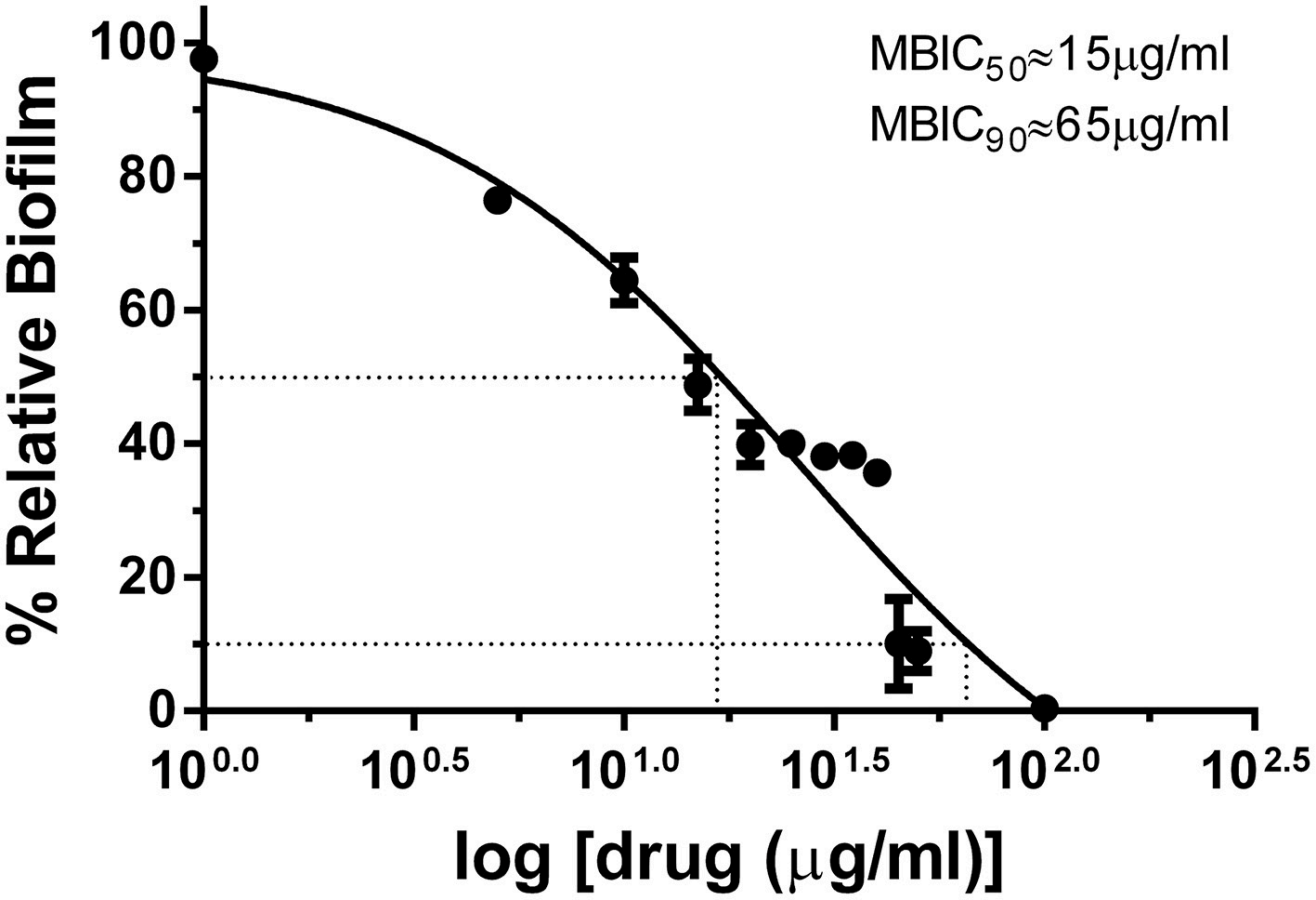
**Effects of varying concentrations of UTI^QQ^ on biofilm of multidrug resistant ***S. aureus*** clinical isolate by crystal violet method**. *S. aureus* clinical isolate (SA13a) was cultured in artificial urine media exposed with varying concentrations of UTI^QQ^ ranging from 1 to 100 μg/mL in triplicates and biofilm was quantified at 595 nm after 24 h by crystal violet method. X-axis represents increasing log concentration of the drug and Y-axis represents the percentage of remaining biofilm compared to the untreated culture control i.e., 100% biofilm. The 50% biofilm inhibition (MBIC_50_) and 90% biofilm inhibition (MBIC_90_) of UTI^QQ^ are 15 and 65 μg/mL, respectively.

**Figure 6:**
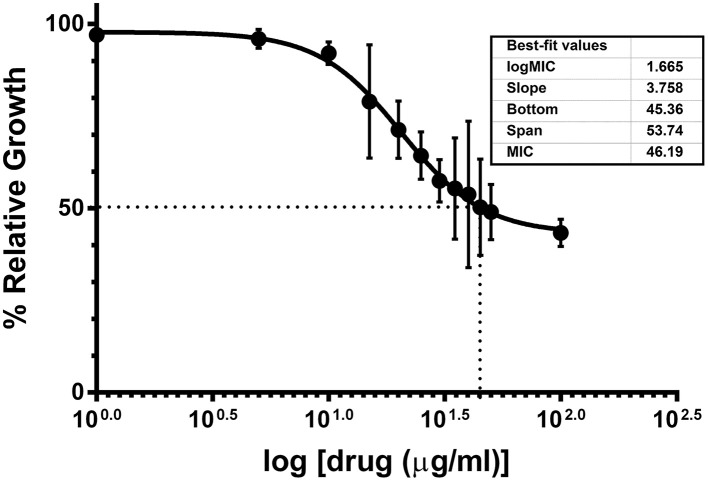
**Effects of varying concentrations of UTI^QQ^ on growth of multidrug resistant ***S. aureus*** clinical isolate**. *S. aureus* clinical isolate (SA13a) was cultured in artificial urine media exposed with varying concentrations of UTI^QQ^ ranging from 1 to 100 μg/mL in triplicates and growth was measured at 600 nm after 24 h. X-axis represents increasing log concentration of the drug and Y-axis represents the percentage of growth compared to the untreated culture control i.e., 100% growth. Growth was reduced to 50% compared to control at around 46 μg/mL concentration of the drug molecule.

**Figure 7:**
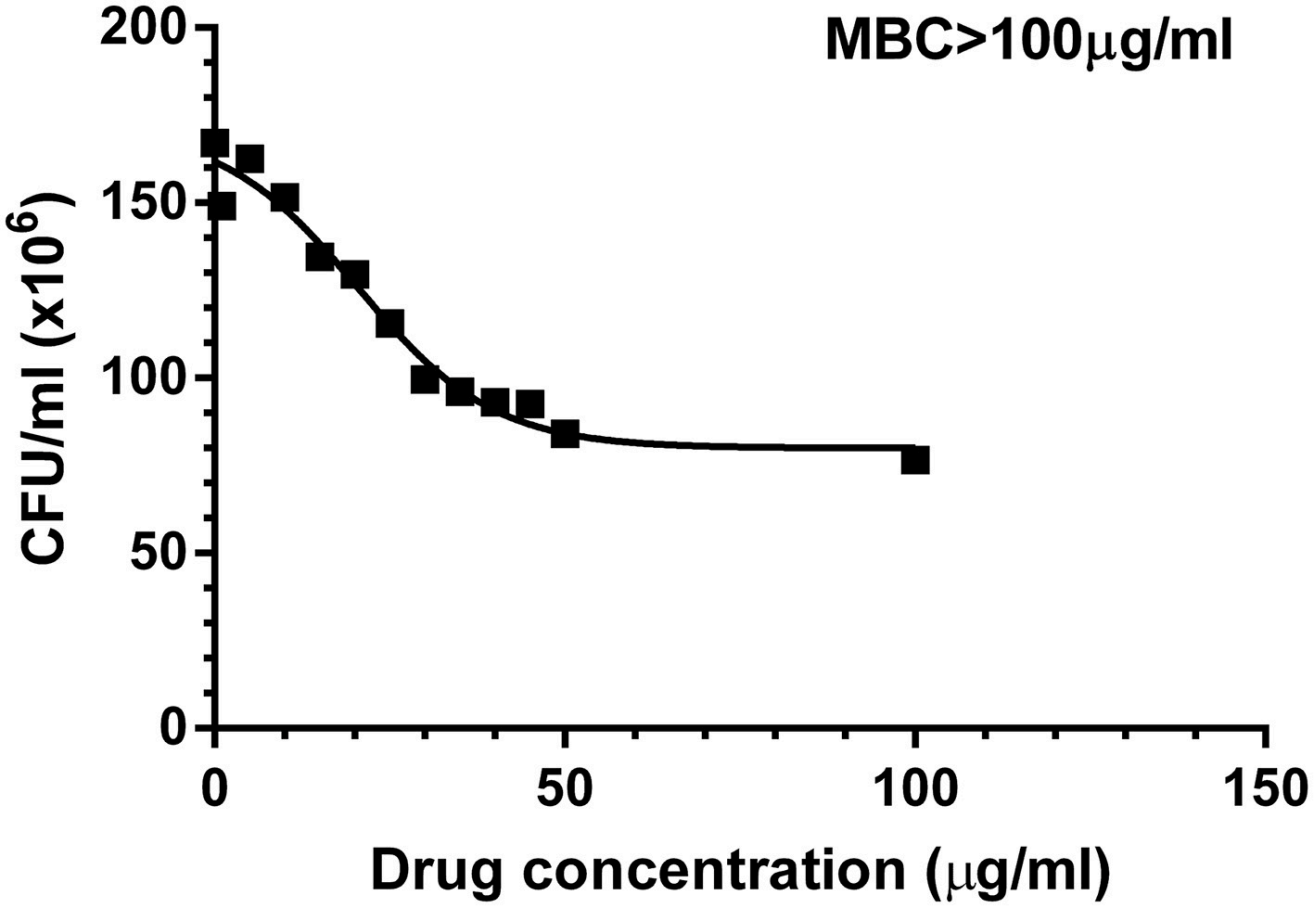
**Effects of varying concentrations of UTI^QQ^ on survival of multidrug resistant ***S. aureus*** clinical isolate**. *S. aureus* clinical isolate (SA13a) treated with varying concentrations of UTI^QQ^ ranging from 1 to 100 μg/mL were plated onto Cation-Adjusted Muller-Hinton Agar (CAMHA) plates. The grown colonies were counted to find the CFU/mL. Survival of bacteria was not much hampered in the range of concentrations tested (1–100 μg/mL) and MBC was greater than 100 μg/mL.

Chemical synthesis of UTI^QQ^ was performed as described before. Yield obtained in reductive amination step was 74% and in esterification step was 58%. The products obtained as a yellow-greenish mixture in the esterification step of reactions was used for the subsequent assays. Minimum biofilm inhibitory concentration of UTI^QQ^ as determined by crystal violet biofilm assay was 15 μg/mL for 50% inhibition (MBIC_50_) and 65 μg/mL for 90% inhibition (MBIC_90_) (Figure 5). This was established by fitting the data obtained on a log (inhibitor) vs. drug dose response curve. Growth was reduced to 50% compared to control at around 46 μg/mL concentration of the drug molecule (Figure 6). It was also observed that the survival of bacteria was not much hampered in the range of concentrations tested (1–100 μg/mL) and MBC was greater than 100 μg/mL (Figure 7). This assay was performed by using the CAMHA plates in triplicates, after appropriate serial dilutions.

**Figure 8:**
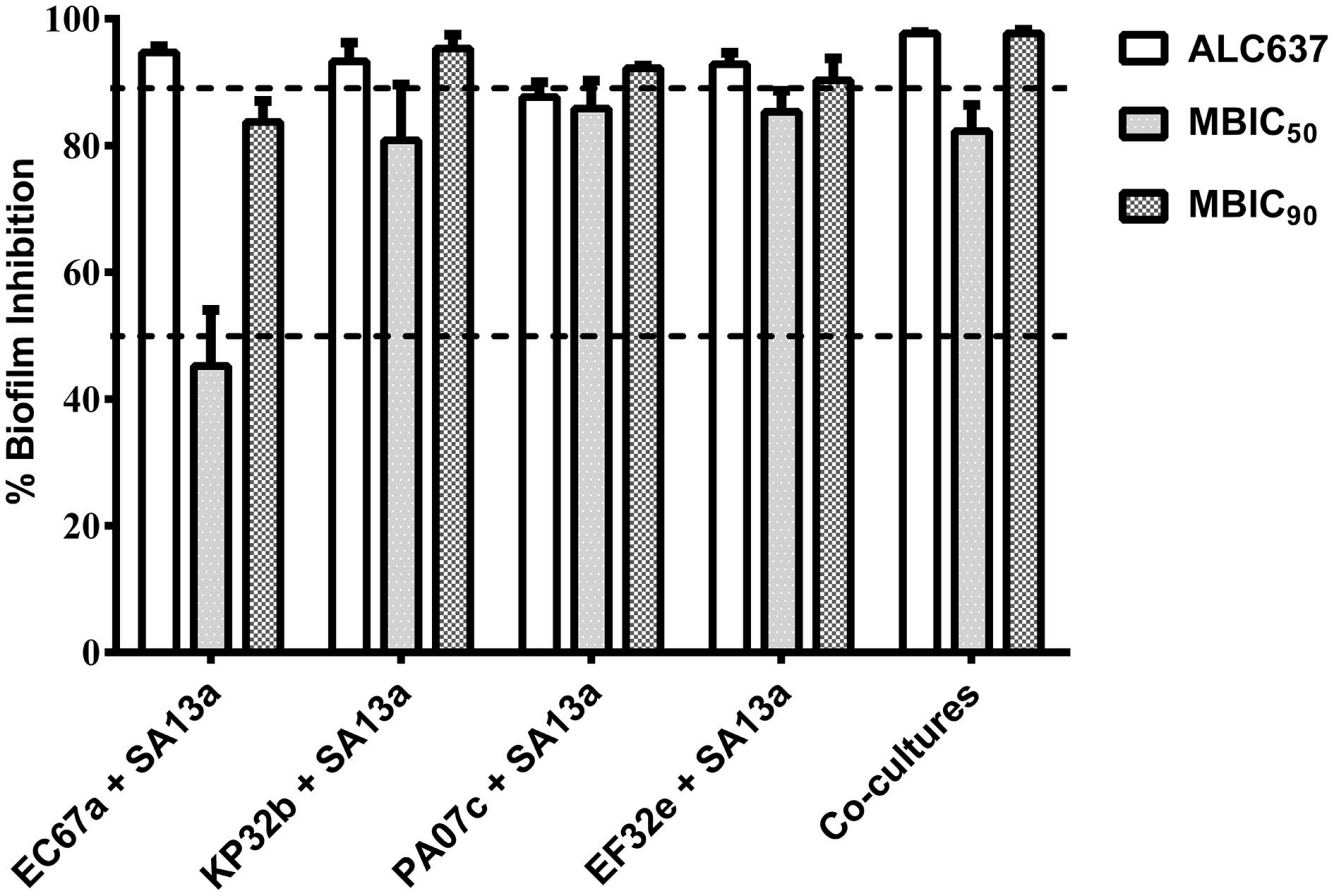
**Effect of UTI^QQ^ on biofilm inhibition in co-culture experiments by crystal violet method**. *S. aureus* clinical isolate (SA13a) was co-cultured with other uropathogens [clinical isolates such as *E. coli* (EC67a), *P. aeruginosa* (PA07c), *K. pneumonia* (KP32b), and *E. faecalis* (EF32e)] and exposed to MBIC_50_ (15 μg/mL) as well as MBIC_90_ (65 μg/mL) of UTI^QQ^. Percentage biofilm inhibition was calculated with that of the untreated co-culture biofilms. *sarA* mutant strain ALC637 (ΔsarA::Tn917LTV1) untreated with UTI^QQ^ was used for comparison of biofilm inhibition by the UTI^QQ^ treated co-cultures. Significant biofilm inhibition was observed in the co-cultures treated with UTI^QQ^ and was comparable to the biofilm of *sarA* mutant.

It was also observed when UTI^QQ^ was used in a co-culture of uropathogens inclusive of *S. aureus*, biofilm reduced significantly in 24 h and reduction was comparable with that of biofilm induced by *sarA* mutant strain *S. aureus* ALC637 (Figure 8). Also there was no significant difference in the biofilm formation in the *sarA* mutant strain (ALC637) when treated with the drug (Figure S2).

### Hydrophobicity assay

**Figure 9:**
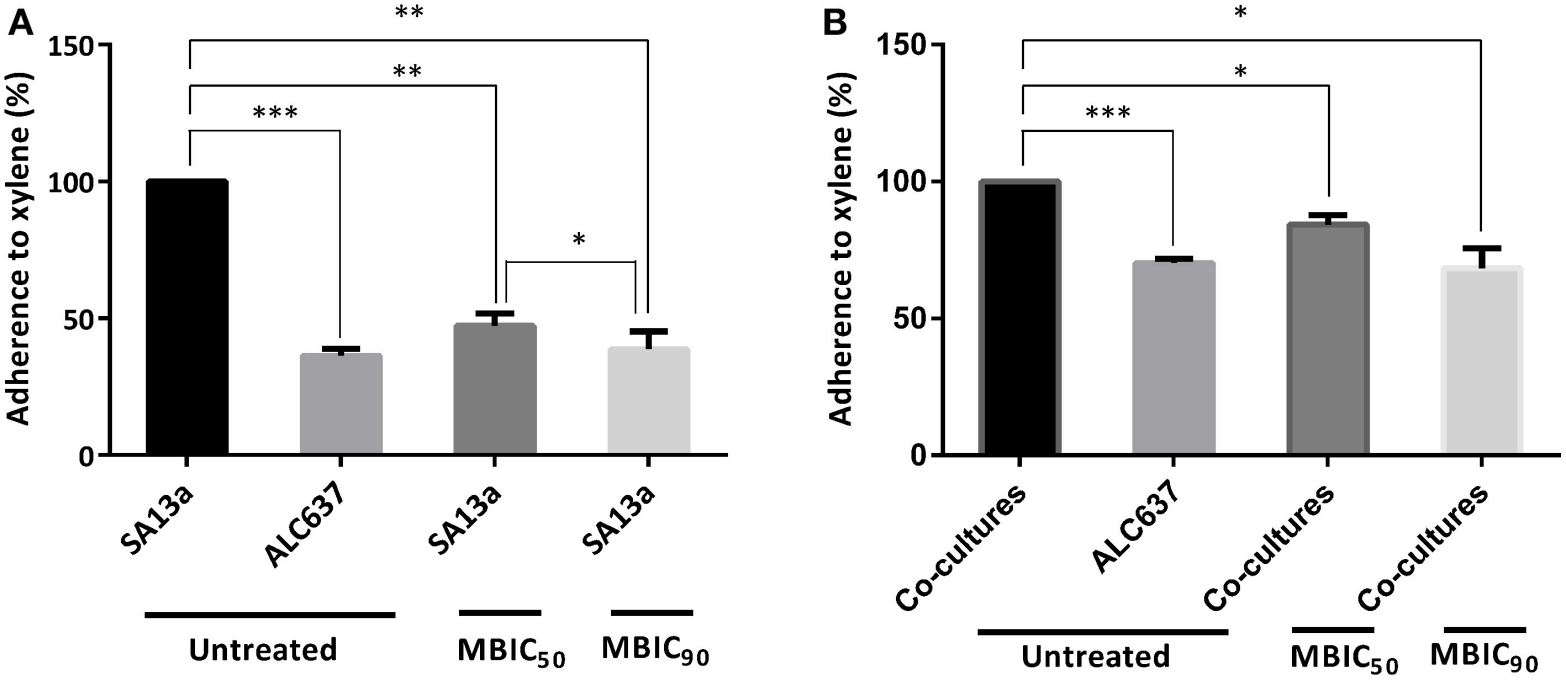
**Hydrophobicity of bacterial cells treated with UTI^QQ^**. **(A)** Percentage adherence of multidrug resistant *S. aureus* clinical isolate (SA13a) to xylene. **(B)** Percentage adherence of co-cultures of uropathogens [clinical isolates such as *E. coli* (EC67a), *S. aureus* (SA13a), *P. aeruginosa* (PA07c), *K. pneumonia* (KP32b) and *E. faecalis* (EF32e)] to xylene. UTI^QQ^ was administered at a concentration of 15 μg/mL (MBIC_50_)and 65 μg/mL (MBIC_90_) to bacterial cultures and the treated cultures were exposed to xylene. UTI^QQ^ untreated bacterial cultures exposed to xylene were kept as control. The difference was statistically tested to check the significance by Multiple comparisons using Tukey's test; Statistical significance ^*^p < 0.05, ^**^p < 0.01, ^***^p < 0.001. Decreased hydrophobicity was observed in the drug treated cultures.

It was found that in comparison with the untreated control, hydrophobicity was reduced by more than 50% in the case of the *S. aureus* clinical isolate monoculture (Figure 9A), or by 30–35% in the case of the equivolume co-culture of uropathogens (Figure 9B).

### Confocal laser scanning microscopy

**Figure 10:**
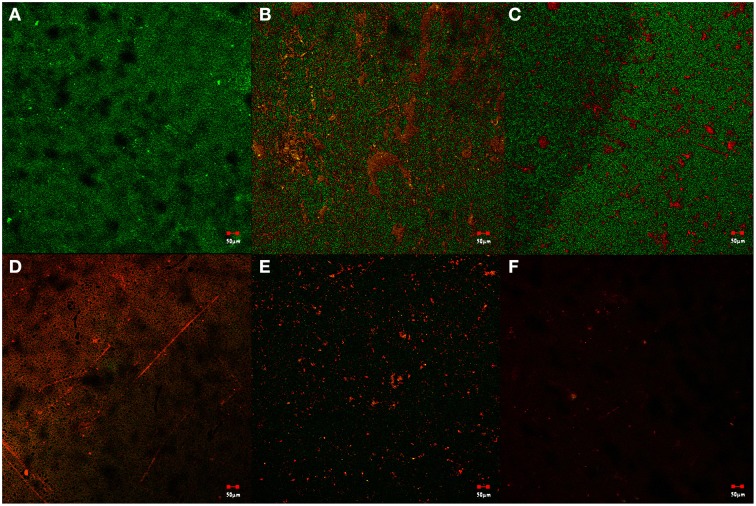
**Live/dead staining observed using confocal laser scanning microscopy**. Biofilm of the mixed bacterial population was developed onto glass cover-slips under various conditions of treatment using SarABI^M^, *o*-coumaric acid, hybrid drug UTI^QQ^ and antibiotic gentamicin. **(A)** Control with no drug treatment, **(B)** SarABI^M^ treatment at MBIC_50−_(40 μg/mL), **(C)** *o*-coumaric acid treatment at MBIC_50_ (5 μg/mL), **(D)** hybrid drug UTI^QQ^ treatment at MBIC_50_ (15 μg/mL), **(E)** Gentamicin treatment at MIC (2 μg/mL), **(F)** Synergetic effects of Gentamicin (2 μg/mL), and UTI^QQ^ (15 μg/mL).

CLSM images of biofilm inhibition caused by the drug treatment were taken as visual confirmation of the drug action (Figure 10). The fixed biofilms were stained using FITC dye which fluoresces green on attachment to the peptides on the surface of healthy cells constituting the biofilm, while the stain EtBr intercalates with the extracellular DNA formed as a result of the lysis of cells and fluoresces red. Untreated cells were taken as the negative control to further visualize the healthy biofilm. Drug treatments with SarABI^M^, *o*-coumaric acid and UTI^QQ^ showed disruption of biofilm, but not much of cell inhibition. Treatment with gentamicin showed cell growth inhibition. When used in combination with UTI^QQ^, the activity of gentamicin had been confirmed to increase significantly with an increased levels of dead cells in the biofilm.

### Cell culture studies

Cell culture studies yielded the LDH value and the MTT assay values required for determining the cytotoxicity of the drug. The LDH concentration reflects the amount of the lysed cells in the medium as it infers the cytotoxicity of the drug. The LDH concentration was observed (Table S2) to be less in drug treated cases when compared to the control as it strongly suggests that the drug has a minimum or no cytotoxic effect.

The Hep-G2 cell viability was also tested using MTT assay which showed the drug, UTI^QQ^ does not affect the viability of the cells (Table S3) complementing the favorable characteristics of the drug. This data significantly provides an insight that the compound neither affects the viability nor toxic to the Hep-G2 cells to have drug-likeness effect.

### *In vivo* studies

**Figure 11:**
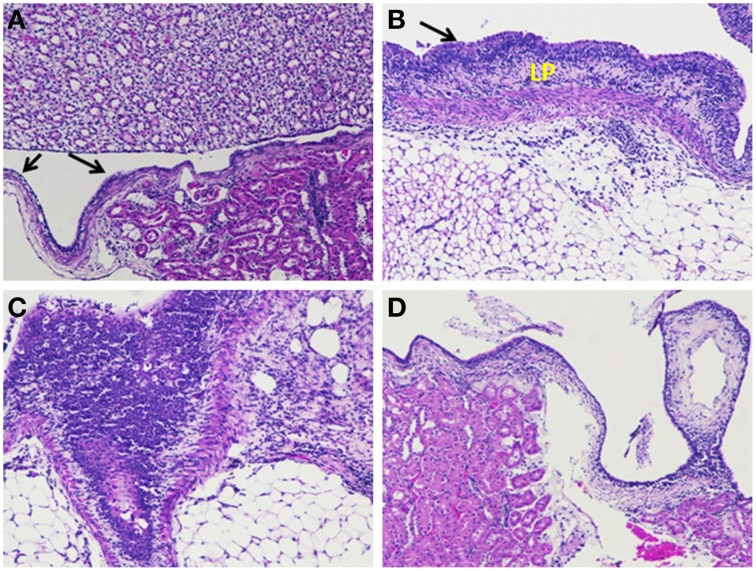
**Histological analyses of kidney tissue sections**. **(A)** Kidney sections of the control group of rat showing normal transitional epithelium–urothelium (arrow) with no significant pathological changes. **(B)** Kidney section of diseased rat inoculated with *S. aureus* revealing moderate hyperplasia of the urothelium (arrow) and abundant lympho-plasmacytic infiltration (LP). **(C)** Kidney section of rat inoculated with *S. aureus* and treated with Gentamicin (LD) having moderate hyperplasia and abundant polymorphonuclear, few lympho-plasmacytic infiltrates beneath the urothelium. **(D)** Kidney section of rat inoculated with *S. aureus* and treated with Gentamicin (HD) having minimal infiltrates, predominantly lymphocytes (Gentamicin, LD, 8 mg/kg and HD, 50 mg/kg).

**Figure 12:**
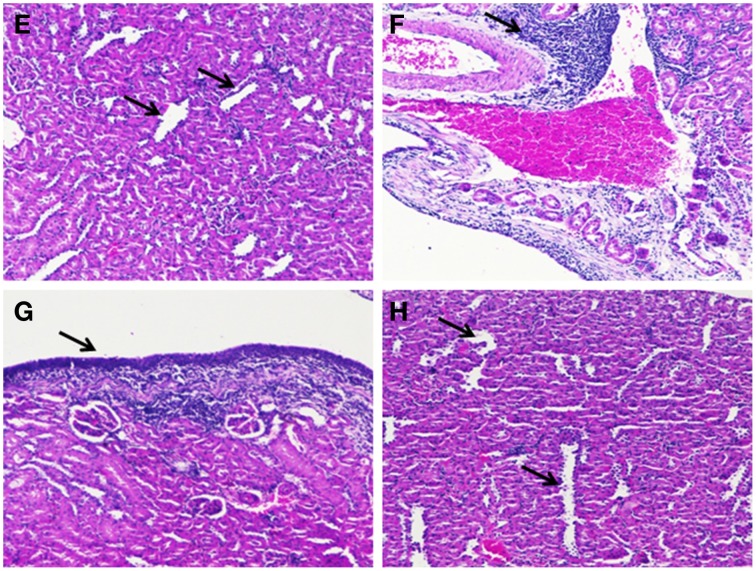
**Histological analyses of kidney tissue sections. (E)** Kidney section of rats inoculated with *S. aureus* and co-treated with the UTI^QQ^ (LD) showing minimal dilatation/degeneration of tubules (arrow). The presence of majority of the tubules in the adjacent areas showed no significant pathological changes. **(F)** Kidney section of the rats inoculated with *S. aureus* and co-treated with - UTI^QQ^ (HD) revealed moderate lympho-plasmacytic cell infiltration (arrow). **(G)** Kidney section of the rat inoculated with *S. aureus* and co-treated with the combinatorial drugs, gentamicin and UTI^QQ^ (LD) showed mild hyperplasia of the urothelium (arrow) and minimal amount of mononuclear cell infiltrates. **(H)** Kidney section of the rats inoculated with *S. aureus* and co-treated with gentamicin and UTI^QQ^ (HD) having minimal dilatation/degeneration of tubules with no significant inflammatory changes. (Gentamicin, LD, 8 mg/kg and HD, 50 mg/kg; Hybrid molecule UTI^QQ^, LD, 16 mg/kg and HD, 64 mg/kg).

*In vivo* studies of gestational UTI in pregnant rats showed no preterm labor symptoms or preterm delivery of pups and the percentage mortality observed was nil (Table S4). Also, the difference of mean body weight of the pups between the control, diseased and the treated groups were not significant (Figure S1). Table 3 shows the viable count of bacteria recovered from tissue the homogenates of kidney. Kidney sections of the control rats appeared histologically normal with no significant pathological changes. Kidney sections of rats inoculated with *S. aureus* had moderate hyperplasia of the urothelium and chronic inflammation of moderate degree in the renal pelvis characterized predominantly by varying degrees of lympho-plasmacytic/polymorphonuclear infiltrates. The transitional epithelium appeared hyperplastic with the presence of a linear thickening of the lining epithelium with lack of no prominent outward or inward growth. In case of Gentamicin (LD) treated groups, a mild reduction in the degree of inflammation was noted and at high dose levels of Gentamicin a moderate decrease in the degree of inflammation was observed. Treatment with UTI^QQ^ at both low and high dose levels caused a mild to moderate reduction in the degree of chronic inflammation associated with the urothelium. Combined treatment of low dose of Gentamicin and UTI^QQ^ resulted in a mild to moderate reduction in the degree of inflammation, whereas the high dose levels had marked a reduction in the degree of inflammation present with few tubules of minimal dilatation/degeneration (Figures 11, 12).

**Table 3:** **Viable count of bacteria recovered from tissue homogenates of kidney**.

**Group**	***S. aureus* Log CFU/g ± SD**
	**kidney**
Control	Nil
Diseased	5.9 ± 0.3
Gentamicin (LD)	6.0 ± 0.1
Gentamicin (HD)	2.0 ± 3.5
UTI^QQ^ (LD)	5.9 ± 0.3
UTI^QQ^ (HD)	3.9 ± 3.4
Gentamicin and UTI^QQ^ (LD)	1.9 ± 3.2
Gentamicin and UTI^QQ^ (HD)	1.9 ± 3.3

*Gentamicin (LD, 8 mg/kg and HD, 50 mg/kg); Hybrid molecule UTI^QQ^ (LD, 16 mg/kg and HD, 64 mg/kg)*.

### Discussion

The threat of MDR appears when a bacterial strain becomes non-responsive to a wide range of antimicrobial concoctions. It is an ever-escalating threat to public healthcare, necessitating the need for the discovery of newer and more potent antibiotics. It is even speculated that with the existing rate of spread of MDR, antibiotics could become obsolete and ineffective. *S. aureus* is one such classical notorious example of MDR pathogen, and it defies attempts of microbial control, in a pattern observed worldwide (Onanuga et al., 2005). Several infections, including that of the urinary tract, can be caused by methicillin-resistant *S. aureus* (MRSA), which is encountered at alarming rates, and this eventually limits the choice of etiological control agents employed to combat infections (Araki et al., 2002; Manikandan et al., 2011). Also, it is an opportunistic pathogen and is known to exhibit immuno-evasive strategies (Kuehnert et al., 2006). Quorum sensing inhibitory approach is a promising alternative to antibiotics which exerts least selective pressure that will possibly prohibit the bacterial strains from attaining resistance (Njoroge and Sperandio, 2009; Defoirdt et al., 2011). In the present investigation, we have developed and evaluated a biofilm inhibitor molecule against multidrug resistant *S. aureus* by *in vitro* and *in vivo* studies.

In our previous studies, we have shown that the root ethanolic extracts of *M. dubia* displayed better antibiofilm activities against *S. aureus* ATCC 25923 (Vinothkannan et al., 2013; Adline Princy et al., 2014). In our another study, we have reported the antibiofilm activity of SarABI against *S. aureus* associated with vascular graft infections (Arya et al., 2015). In the present study developing a hybrid drug molecule (here referred as UTI^QQ^) to posess better activity was attempted in light of the results from our previous studies. Initially, GC-MS analysis followed by computational studies scored the top-hit molecules on applying the principles of druglikeliness and among the first four top-hits were found to have sugar moeity and hence exhibited low druglikeliness. On the other hand, the fifth molecule, although an acid, has a positive logP and fits into Lipinski's rule of five (Lipinski et al., 2012). The molecule bears the chemical name 2-hydroxycinnamic acid, commonly termed as *o*-coumaric acid (Table 2). Antioxidant properties have been reported previously in both *o*-coumaric acid (Ferguson et al., 2005) and in the more abundant isomer *p*-coumaric acid (Elfalleh et al., 2011), which is found in lignin and as a constituent of pollen honey and vinegar. *O*-coumaric acid was found to have interactions with R84 and R90 (Figure 3). Since the acids qualify rarely as drugs (accepted only as salts or other derivatives), an attempt was evoked to hybridize the acid with an earlier established quorum quencher of SarA, namely the SarABI. The ester between the two was predicted to have a low binding score of −4.351, but the ester (UTI^QQ^) formed between modified form of SarABI and *o*-coumaric acid was predicted to have binding sites on R84, R90 and Q64, and a G-score of −5.151 (Figure 4).

The hybrid drug molecule, UTI^QQ^ was then chemically sysnthesized and validated. Quantitative biofilm assay results by crystal violet method suggested that the biofilm of *S. aureus* clinical isolate (SA13a) and biofilm of mixed species of clinical isolates of uropathogens got inhibited in the presence of UTI^QQ^. This in turn suggest that the interspecies cooperation hinging on *S. aureus* is instrumental to infections. The co-culture biofilm results suggests that elimination of *S. aureus* biofilm leads to the collapse of the mixed species biofilm as well, caused as a result of depreciating ecological fitness. Hence, UTI^QQ^ can potentially be used to treat all UTI infections involving *S. aureus*, irrespective of whether it remains the major perpetrator or not. In addition, no significant difference in the biofilm reduction was observed between the UTI^QQ^ treated and untreated of *sarA* mutant, ALC637 (Figure S2). The result also suggest the mode of action of the drug on downregulating *S.aureus* adherence via., negatively regulating the quorum regulator, SarA as envisioned by the docking studies.

The hydrophobicity of the cells were determined by partitioning the cells between water and xylene, and the measure of adherence to xylene gave a measure of hydrophobicity of the cell surface components. The strong biofilm producers were always highly hydrophobic in nature. In the present study the strains treated with UTI^QQ^ showed to exhibit less biofilm forming capabilities with low hydrophobic nature.

CLSM analysis showed mixed populations of UTI pathogens have been shown to be hit hard by the presence of the UTI^QQ^. This is indicative of the suitability of the drug against UTIs involving *S. aureus*. Further, the drug, when used in combination with the broad spectrum antibiotic gentamicin, resulted increased activity of gentamicin. This implies that the drug could well be used along with gentamicin at a lower concentration, and be applied to potentiate antibiotic action as an additive effect. The mode of action of UTI^QQ^ is that by destabilizing the biofilm, exposure of cells to external agents (such as gentamicin in this case) ensues. When the biofilm is dissipated, the ecosystem of mixed populations fails, in turn triggering the halt of the infestation. This makes it undemanding for the antibiotic to penetrate the biofilm and actuate its bactericidal effects.

In a study involving mice, gestational UTI was identified to be a cause of miscarriages and premature births (Kaul et al., 1999). Our *in vivo* results are in contrast with Kaul et al. (1999) where they had observed a significant difference in the percentage mortality of the delivered pups from *E. coli* infected mice. We have not observed any miscarriages or premature births in the infected rats. However, our quantitative tissue culture results rules out the possibility of unsuccessful experimental UTI since the recovery of uropathogenic *S. aureus* from kidney samples was achieved (Table 3). In addition, the retainment of pathogenic virulence in our uropathogen was clearly seen from the kidney histological sections of the diseased group showing inflammation (Figure 11). Reduced colony counts and mild inflammation in the combined treatment of gentamicin and UTI^QQ^ (Table 3 and Figure 12) suggests again that UTI^QQ^ could give an addictive effect to gentamicin action like as shown in our CLSM studies (Figure 10). Overall, the *in vitro* and *in vivo* experimental data suggests that the UTI^QQ^ alone or, in combination with gentamicin (in our study) could be a possible therapy for staphylococcal associated UTI.

## Conclusions

Urinary tract infections by *S. aureus* are among the most difficult-to-treat ailments during pregnancy. This is partly due to the inability of physicians to prescribe certain broad spectrum antibiotics, and partly due to the increasingly alarming trend of pathogens acquiring drug resistance within quick span of time. Quorum quenching is a neoteric approach to combat sequelae arising from bacterial infections. Antivirulence drug therapy aiming to disarm the pathogens is often advantageous mainly because such drugs work even in resistant strains where antibacterial or bactericidal drugs fail. Drugs that target the interbacterial communication are being explored in a bid to stall the tremendous rate of resistance evolution. Plant constituents have often had molecules that hinder the growth of bacteria and this explains why human pathogens do not colonize plants.

In this study, various molecules identified from *Melia dubia* has been screened for suitability for being used as an antagonist against an important transcriptional factor that regulates virulence and biofilm formation. 2-hydroxycinnamic acid turned out to be the most probable cause of antibiofilm activity of *M. dubia* root ethanolic extracts and hence was taken up for validation as an ester with 4-benzylamino cyclohexanol. This concept of hybrid molecule arises when both the pharmacophores are capable of individual activity, and enhanced activity when fused into a single pharmacophore molecule. *In vitro* studies have established unquestionably the activity of the drug. Cell culture studies have also demonstrated the suitability of such a drug on real time in living systems. By destabilizing the biofilms involving *S. aureus*, we can script the collapse of the ecosystem of the causal mixed species pathogens and ultimately stall the invasion. A decisive application of this drug would be to render the pathogens more vulnerable to antibiotic action and thus eliminate the illness at lower concentrations of antibiotic, when used in combination with the quorum blockers.

## Author contributions

All the authors have equally contributed to the manuscript.

### Conflict of interest statement

The authors declare that the research was conducted in the absence of any commercial or financial relationships that could be construed as a potential conflict of interest.

## Supplementary material

Figure S1 **Mean body weight of the delivered pups from gestational UTI pregnant rats**. Differences of the mean body weight of the pups between the control, diseased and the treated groups were not significant. Gentamicin (LD, 8 mg/kg and HD, 50 mg/kg); Hybrid molecule UTI^QQ^ (LD, 16 mg/kg and HD, 64 mg/kg).

Figure S2 **Percentage biofilm inhibition of ***sarA*** mutant ALC637 (ΔsarA::Tn917LTV1) on treatment with UTI^QQ^ by crystal violet method**. Wild-type and* sarA* mutant strain of *S. aureus* were UTI^QQ^ treated with a concentration of 15 μg/mL (MBIC_50_)and 65 μg/mL (MBIC_90_). UTI^QQ^ treated cultures were compared with that of untreated *S. aureus* clinical isolate, SA13 (Negative control). No significant difference in the biofilm reduction was observed between the treated and untreated *sarA* mutant, ALC637.

Table S1 **List of ligands obtained from ***Melia dubia*** root extract by GC-MS analysis**.

Table S2 **LDH concentration of untreated Hep-G2 cells and cells treated with UTI^QQ^**.

Table S3 **MTT assay values of untreated Hep-G2 cells and cells treated with UTI^QQ^**.

Table S4 **Percentage mortality of pups delivered after the experimental UTI induced with uropathogenic ***S. aureus*** in wistar rats**.
