# Supplementary material for: Development of a biofilm inhibitor molecule against multidrug resistant Staphylococcus aureus associated with gestational urinary tract infections
Source: Front Microbiol. 2015 Aug 11;6:832. doi: 10.3389/fmicb.2015.00832 (PMC4531255; doi:10.3389/fmicb.2015.00832)
Supplement: Table S1 — List of ligands obtained from Melia dubia root extract by GC-MS analysis. [file Table1.DOC]

**Table S1 List of ligands obtained from *Melia dubia* root extract by GC-MS analysis.**

| **S. No.** | **Peak name** | **Retention time (min)** |
| --- | --- | --- |
| 1 | Glycerin | 6.7 |
| 2 | Propanoic acid, 2-oxo-, methyl ester | 9.99 |
| 3 | Octanediamide, N,N'-di-benzoyloxy- | 10.52 |
| 4 | Piperazine, 1-(aminoacetyl)- | 11.25 |
| 5 | Dianhydromannitol | 11.59 |
| 6 | 2-Butanone, 4-phenyl- | 12.03 |
| 7 | L-Galactose, 6-deoxy- | 12.31 |
| 8 | 2-Nonen-1-ol | 13.43 |
| 9 | Cyclohexanecarboxylic acid, 3-(acetyloxy) | 15.03 |
| 10 | 5,6-Epoxy-6-methyl-2-heptanone | 15.14 |
| 11 | Vanillin lactoside | 15.22 |
| 12 | Sucrose | 15.88 |
| 13 | 2-hydroxycinnamic acid / *o*-coumaric acid | 16.13 |
| 14 | 1,7-Octanediol, 3,7-dimethyl- | 16.4 |
| 15 | Hexanoic acid, 2-methyl- | 17.19 |
| 16 | Benzene, 1,2,3-trimethoxy-5-(2-propenyl)- | 17.68 |
| 17 | Undecanoic acid | 17.88 |
| 18 | Benzenepropanol, 4-hydroxy-à-methyl-, (R)- | 18.19 |
| 19 | Ethyl à-d-glucopyranoside | 18.91 |
| 20 | 1-Cyclohexanol, 1-[5-hydroxy-4-methyl-2-hexenyl] | 19.34 |
| 21 | d-Mannose | 19.43 |
| 22 | [1,1'-Bicyclopropyl]-2-octanoic acid, 2'-hexyl-, methyl ester | 19.74 |
| 23 | 3,7,11-Trimethyl-dodeca-2,6,10-trienoic acid | 19.87 |
| 24 | 1,2-15,16-Diepoxyhexadecane | 20.01 |
| 25 | 4-Cyclononen-1-one | 20.27 |
| 26 | Decanoic acid, 3-methyl- | 20.39 |
| 27 | 2-Butyl-5-methyl-3-(2-methylprop-2-enyl)cyclohexanone | 21.13 |
| 28 | Tetradecanoic acid, ethyl ester | 21.35 |
| 29 | 2-Indanone, 4,5,6,7-tetrahydro- | 23.26 |
| 30 | n-Hexadecanoic acid | 23.69 |
| 31 | 2-Cyclohexen-1-one, 2-(2-methyl-2-propenyl)- | 24.46 |
| 32 | (E)-9-Octadecenoic acid ethyl ester | 26.12 |
| 33 | 3,7,7-Trimethyl-1-penta-1,3-dienyl-2-oxabicyclo[3.2.0]hept-3-ene | 26.47 |
| 34 | Menthol, 1'-(butyn-3-one-1-yl)-, (1S,2S,5R)- | 26.78 |
| 35 | 2-(3,4-Methylenedioxyphenyl)cyclohexanone | 28.45 |
| 36 | Hexadecanoic acid, 2-hydroxy-1-(hydroxymethyl)ethyl ester | 30.19 |
| 37 | Hexadecanoic acid, ethyl ester | 31.16 |
| 38 | Docosanoic acid, ethyl ester | 31.74 |
| 39 | Stigmasterol | 32.97 |
| 40 | Cholesta-22,24-dien-5-ol, 4,4-dimethyl- | 33.79 |
