# Supplementary material for: Development of a biofilm inhibitor molecule against multidrug resistant Staphylococcus aureus associated with gestational urinary tract infections
Source: Front Microbiol. 2015 Aug 11;6:832. doi: 10.3389/fmicb.2015.00832 (PMC4531255; doi:10.3389/fmicb.2015.00832)
Supplement: Table S2 — LDH concentration of untreated Hep-G2 cells and cells treated with UTIQQ. [file Table2.DOC]

**Table S2 LDH concentration of untreated Hep-G2 cells and cells treated with UTI^QQ^.**

| **Assay** | **Control (without drug)** | **MBIC_50_**  **(15 µg/ml)** | **MBIC_90_**  **(65 µg/ml)** |
| --- | --- | --- | --- |
| LDH Assay | 0.920 IU/L | 0.638 IU/L | 0.754 IU/L |
