# Supplementary material for: Development of a biofilm inhibitor molecule against multidrug resistant Staphylococcus aureus associated with gestational urinary tract infections
Source: Front Microbiol. 2015 Aug 11;6:832. doi: 10.3389/fmicb.2015.00832 (PMC4531255; doi:10.3389/fmicb.2015.00832)
Supplement: Table S3 — MTT assay values of untreated Hep-G2 cells and cells treated with UTIQQ. [file Table3.DOC]

**Table S3 MTT assay values of untreated Hep-G2 cells and cells treated with UTI^QQ^.**

| **Time (h)** | **Control (without drug)** | **MBIC_50_**  **(15 µg/ml)** | **MBIC_90_**  **(65 µg/ml)** |
| --- | --- | --- | --- |
| 0 | 0.361 | 0.450 | 0.425 |
| 1 | 0.546 | 0.816 | 0.798 |
| 2 | 0.689 | 0.868 | 1.007 |
| 3 | 0.721 | 0.957 | 1.118 |
