# Supplementary material for: Development of a biofilm inhibitor molecule against multidrug resistant Staphylococcus aureus associated with gestational urinary tract infections
Source: Front Microbiol. 2015 Aug 11;6:832. doi: 10.3389/fmicb.2015.00832 (PMC4531255; doi:10.3389/fmicb.2015.00832)
Supplement: Table S4 — Percentage mortality of pups delivered after the experimental UTI induced with uropathogenic S. aureus in wistar rats. [file Table4.DOC]

**Table S4 Percentage mortality of pups delivered after experimental UTI with uropathogenic *S. aureus*** in wistar rats.

| **Group** | **No. of rats (%)** |  |  | **Day of**  **delivery** | **No. of rats delivering** | **% Mortality of pups born (no. died/ total no.)** |
| --- | --- | --- | --- | --- | --- | --- |
|  | **Total** | **Preterm** | **Term** |  |  |  |
| Control | 3 (100) | 0 (0) | 3 (100) | 22  23 | 2  1 | 0 (0/19) |
| Diseased | 3 (100) | 0 (0) | 3 (100) | 22  24 | 2  1 | 0 (0/19) |
| Gentamicin (LD) | 3 (100) | 0(0) | 3(100) | 22  26 | 2  1 | 0 (0/26) |
| Gentamicin (HD) | 3 (100) | 0(0) | 3(100) | 23  22 | 2  1 | 0 (0/35) |
| UTI^QQ^ (LD) | 3 (100) | 0(0) | 3(100) | 25  26 | 2  1 | 0 (0/34) |
| UTI^QQ^ (HD) | 3 (100) | 0(0) | 3(100) | 22  23  25 | 1  1  1 | 0 (0/29) |
| GEN and UTI^QQ^ (LD) | 3 (100) | 0(0) | 3(100) | 22  24 | 2  1 | 0 (0/18) |
| GEN and UTI^QQ^ (HD) | 3 (100) | 0(0) | 3(100) | 22  23 | 2  1 | 15.8 (3/19) |

Gentamicin (LD - 8 mg/kg and HD - 50 mg/kg); Hybrid molecule UTI^QQ^ (LD - 16 mg/kg and HD - 64 mg/kg)
